# The Value of Abdominal Palpation in Labour.—II

**Published:** 1909-05-01

**Authors:** 


### Obstetrics.

### THE VALUE OF ABDOMINAL PALPATION IN LABOUR.—II.

The value of abdominal palpation for the diagnosis of positions of the fœtus other than the uncomplicated vertex, breech, or transverse, is not fully realised, and the amount of information to be obtained from it in abnormal cases can only be appreciated by those who constantly practise it. In face presentations, for instance, the signs elicited by abdominal palpation are usually quite diagnostic, and require no confirmation by vaginal touch.

The pelvis is empty at the beginning of labour, or the pelvic brim is only partially filled by the presenting part. This occurs because the face does not sink into the brim before labour begins, as the vertex does. At the same time there will be no difficulty in diagnosing a cephalic lie of the fœtus, because the head can be felt and perhaps moved just at the brim. In favourable cases the chin can be positively felt dipping into the pelvis, whilst the occiput stands out hard and rounded with a distinct groove between it and the fœtal back. The spine of the fœtus may be found extended or hyperextended instead of being arched in flexion, and the fœtal heart sounds are best heard on the side at which the limbs are felt. This no doubt depends on extension of the fœtal spine, which naturally brings the ventral surface of the fœtus nearer to the uterine wall.

It is needless to say that the early recognition of a face presentation may be of the greatest importance, for in the absence of contraction of the pelvis or other obstructing element, early recognition will permit of an attempt to change the presentation into that of the vertex. This cannot be done with much chance of success when the face has already sunk into the pelvic cavity. It is in mento-posterior positions of the face that the child runs the greatest risk from the length of labour and the possibility of non-rotation of the chin, but even in mento-anterior cases the child is much more likely to suffer than in any vertex position.

Contraction of the pelvis, both in major and minor degrees, is often overlooked in primiparæ until labour has been in progress many hours. This is a very serious business for the child, and may be equally so to the mother; yet such an accident cannot happen if a careful examination is made at the beginning of labour. The first point which is usually striking is the prominence of the abdomen combined with tenseness of the abdominal walls. This is especially the case in short women. Next it will be noted that the fœtal head if presenting is not engaged, and is movable from side to side above the brim. This point alone in a primipara indicates either an abnormal presentation or something which interferes with the entrance of the head into the brim. Some form of contracted pelvis is by far the commonest cause of such mobility of the head above the brim in a primipara.

In a multipara the head may be movable above the brim on account of a pendulous condition of the abdomen, but the previous history will then either negative or corroborate the diagnosis of contracted pelvis.

The position of the head in a flat pelvis is often transverse, as the head naturally tries to enter the brim in the widest diameter, and further flexion will usually be incomplete in such a case, so that forehead and occiput will be on the same level. These signs will often indicate a contracted pelvis when it is impossible to make the diagnosis by vaginal examination. In a primipara it may be impossible to reach the promontory of the sacrum except when under chloroform, and yet there may be an appreciable degree of pelvic contraction. Whilst it is usually possible to confirm the diagnosis of a flat pelvis by direct measurement of the diagonal conjugate the differentiation of a generally contracted pelvis is often extremely difficult. The position in which the head attempts to engage will give some indication of this, for in flat pelves the head engages transversely and a little extended on the fœtal trunk, whilst in generally contracted pelves the head engages in an oblique diameter and in extreme flexion.

It is of some importance to differentiate these two forms of contracted pelvis, because the treatment is essentially different where only minor degrees of contraction are present. In some cases of flat pelvis version gives a distinct advantage if performed at the right time, whilst in generally contracted pelves it may be accepted as a truism that version is absolutely forbidden, and the forceps will offer the best means of delivery. In major degrees of contraction neither of these methods come into consideration.

Again, if induction of labour is determined upon for a minor degree of contracted pelvis, then abdominal palpation is the only means we have of deciding the exact moment at which labour should be induced. As long as the fœtal head can be easily pushed down into the pelvis the pregnancy may be allowed to proceed, and labour should be induced only when the head can be pushed down with difficulty. It has not seldom occurred that labour has been induced too early, and a small puny child, with very little chance of survival, has been born, a result of an easy labour. To instance the discovery of breech presentations, of tumours such as ovarian cysts or uterine fibroids, or of contraction of the pelvis, is to allude to the commoner conditions only which cause abnormal labours.

If possible a breech presentation in a primipara should always be converted into a vertex presentation by external version, and this can best be done at about the end of the seventh month. Later it may be possible, but it can only be very rarely accomplished at the beginning of labour. There are many other conditions which can be elucidated by abdominal palpation; those mentioned have been merely those of the greatest practical importance.

